# DNA methylation of skeletal muscle function‐related secretary factors identifies FGF2 as a potential biomarker for sarcopenia

**DOI:** 10.1002/jcsm.13472

**Published:** 2024-04-20

**Authors:** Jia‐Wen Li, Zheng‐Kai Shen, Yu‐Shuang Lin, Zhi‐Yue Wang, Mei‐Lin Li, Hui‐Xian Sun, Quan Wang, Can Zhao, Jin‐Shui Xu, Xiang Lu, Wei Gao

**Affiliations:** ^1^ Department of Geriatrics, Zhongda Hospital, School of Medicine Southeast University Nanjing China; ^2^ Jiangsu Province Center for Disease Control and Prevention Nanjing China; ^3^ Department of Geriatrics, Sir Run Run Hospital Nanjing Medical University Nanjing China

**Keywords:** DNA methylation, FGF2, Older adults, Sarcopenia

## Abstract

**Background:**

Sarcopenia is characterized by progressive loss of muscle mass and function due to aging. DNA methylation has been identified to play important roles in the dysfunction of skeletal muscle. The aim of our present study was to explore the whole blood sample‐based methylation changes of skeletal muscle function‐related factors in patients with sarcopenia.

**Methods:**

The overall DNA methylation levels were analysed by using MethlTarget™ DNA Methylation Analysis platform in a discovery set consistent of 50 sarcopenic older adults (aged ≥65 years) and 50 age‐ and sex‐matched non‐sarcopenic individuals. The candidate differentially methylated regions (DMRs) were further validated by Methylation‐specific PCR (MSP) in another two independent larger sets and confirmed by pyrosequencing. Receiver operating characteristic (ROC) curve analysis was used to determine the optimum cut‐off levels of fibroblast growth factor 2 (FGF2)_30 methylation best predicting sarcopenia and area under the ROC curve (AUC) was measured. The correlation between candidate DMRs and the risk of sarcopenia was investigated by univariate analysis and multivariate logistic regression analysis.

**Results:**

Among 1149 cytosine‐phosphate‐guanine (CpG) sites of 27 skeletal muscle function‐related secretary factors, 17 differentially methylated CpG sites and 7 differentially methylated regions (DMRs) were detected between patients with sarcopenia and control subjects in the discovery set. Further methylation‐specific PCR identified that methylation of fibroblast growth factor 2 (FGF2)_30 was lower in patients with sarcopenia and the level was decreased as the severity of sarcopenia increased, which was confirmed by pyrosequencing. Correlation analysis demonstrated that the methylation level of FGF2_30 was positively correlated to ASMI (*r* = 0.372, *P* < 0.001), grip strength (*r* = 0.334, *P* < 0.001), and gait speed (*r* = 0.411, *P* < 0.001). ROC curve analysis indicated that the optimal cut‐off value of FGF2_30 methylation level that predicted sarcopenia was 0.15 with a sensitivity of 84.6% and a specificity of 70.1% (AUC = 0.807, 95% CI = 0.756–0.858, *P* < 0.001). Multivariate logistic regression analyses showed that lower FGF2_30 methylation level (<0.15) was significantly associated with increased risk of sarcopenia even after adjustment for potential confounders including age, sex, and BMI (adjusted OR = 9.223, 95% CI: 6.614–12.861, *P* < 0.001).

**Conclusions:**

Our results suggest that lower FGF2_30 methylation is correlated with the risk and severity of sarcopenia in the older adults, indicating that FGF2 methylation serve as a surrogate biomarker for the screening and evaluation of sarcopenia.

## Introduction

Sarcopenia is one of the common geriatric syndromes characterized by progressive loss of skeletal muscle mass accompanied by decline in muscle strength and/or reduced physical performance with advancing age.^1^ The prevalence of sarcopenia ranges from 5% to 13% in the elderly aged 60–70 years and may even increase to approximately 50% in octogenarians.^1^ It has been recognized that sarcopenia is associated with increased risk of falls, fracture, poor quality of life, physical disabilities, and mortality.^1^ Sarcopenia has therefore gradually become one of the major public health problems in the older adults.

To date, the diagnosis of sarcopenia is based on the measurements of muscle mass, muscle strength, and physical performance.^1^ However, due to the relatively high cost and complexity of the examinations, the accuracy and timeliness of the assessment for sarcopenia are limited.^2^ Although considerable efforts have been made in the search for early and convenient tools to aid the diagnosis of sarcopenia,^3^ no valid and specific biomarker has been identified. Thus, the emerging priority is to identify potential biomarkers for the early screening of the elderly at high risk of sarcopenia.

Sarcopenia is a complex multifactorial disorder related to both intrinsic genetic landscape and extrinsic environmental influences.^3^ The mechanisms that involved in the development of sarcopenia include neuromuscular degeneration, stem cells exhaustion, muscle fibre denervation, mitochondrial dysfunction, oxidative stress, and inflammation, and so on.^4^ Besides the genetic aspect, muscular phenotypes are also affected by multiple external factors such as nutrients and physical activity, which might regulate the production and secretion of myokines through epigenetic mechanism.^5^ DNA methylation, which mainly corresponds to the covalent addition of a methyl group to the 5′ position of cytosine in a cytosine‐phosphate‐guanine (CpG) dinucleotide, is one of the best characterized epigenetic modifications in the context of aging.^5^ During the last decades, extensive work has been carried out to link the epigenetic changes to age‐related phenotypes, including sarcopenia.^5^ Although changes of DNA‐methylation in skeletal muscle was found in patients with sarcopenia,^6^ no significant difference in the patterns of DNA methylation was observed in whole blood sample.^7^ In particular, the DNA methylation status in key genes involved in sarcopenia remains unclear. Myokines, secreted by skeletal muscle, play crucial roles in the regulation of muscle contraction and muscle strength.^8^ Previous studies have demonstrated the secretory disturbance of myokines, such as myostatin,^9^, ^10^ irisin,^11^ growth differentiation factor 15,^12^ brain‐derived neurotrophic factor,^13^ secreted protein acidic and rich in cysteine,^14^ apelin,^15^ and so on, during the pathogenesis of sarcopenia. Methylation‐specific PCR (MSP) is one of the most convenient, effective, and rapid methods for examining the methylation status of the promoter regions of individual gene and has been applied for the diagnosis of various human diseases.^16^ Therefore, the present study sought to identify DNA methylation changes in skeletal muscle function‐related secretary factors associated with sarcopenia. In order to facilitate the application of non‐invasive diagnostic tools for sarcopenia, we focused on the blood‐detectable factors and analysed the whole blood sample‐based methylation via differences between sarcopenic and non‐sarcopenic populations.

## Methods

### Study design

**Figure 1:**
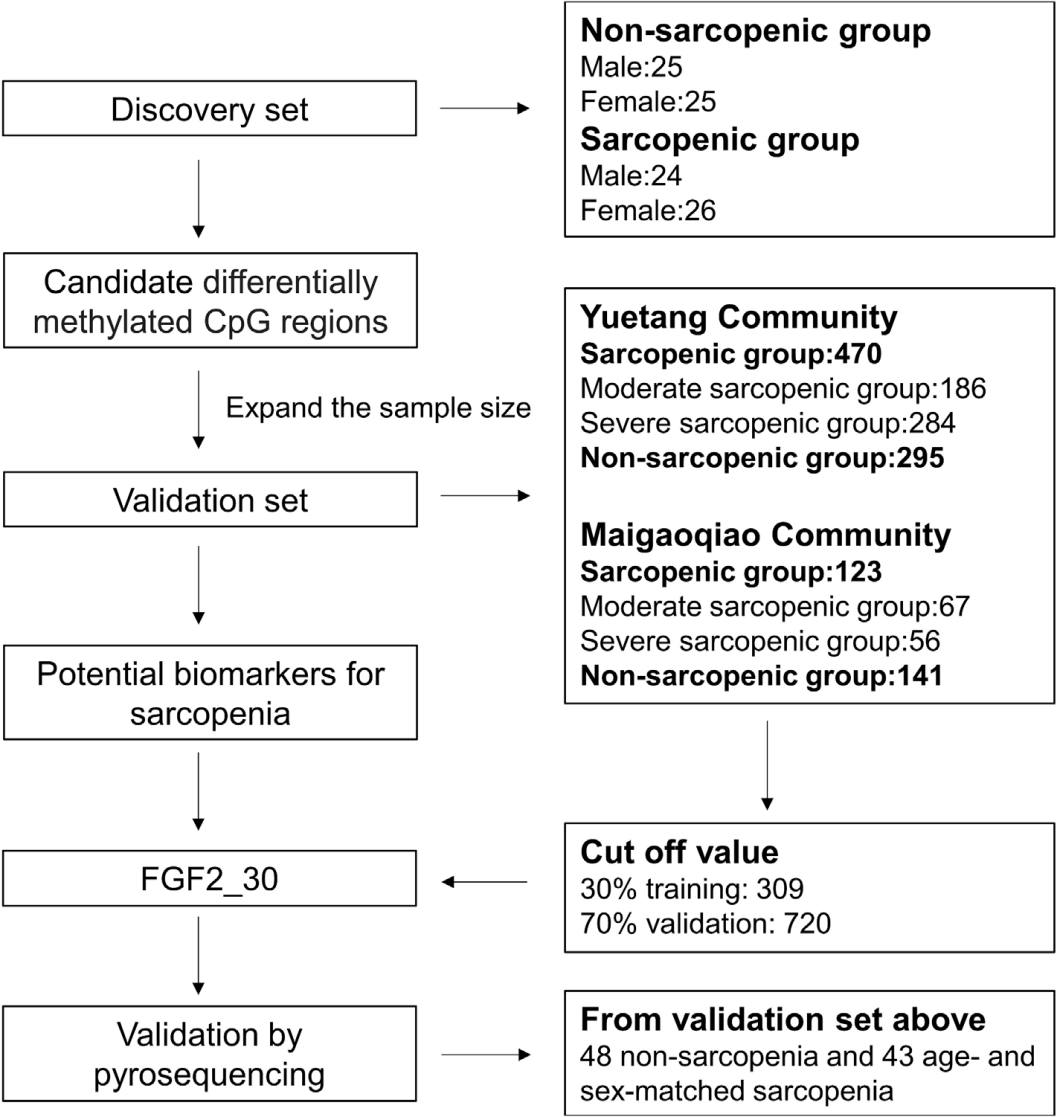
Flowchart of the present study.

For the discovery set, 50 sarcopenic older adults (aged ≥65 years) and 50 age‐ and sex‐matched non‐sarcopenic individuals were selected from the National Basic Public Health Project in Yuetang Community Medical Center in Yangzhou, Jiangsu Province, China. The overall DNA methylation levels were analysed in a set of 27 blood‐detectable secretary factors that have been reported to be crucial for the regulation of skeletal muscle function (detailed information are shown in Table S1). The candidate differentially methylated regions (DMRs) were further validated in a larger set included 470 patients with sarcopenia and 295 control older adults from Yuetang Community Medical Center. The FGF2_30 methylation level was further validated in another set included 123 patients with sarcopenia and 141 control older adults from the National Basic Public Health Project in Maigaoqiao Community Medical Center in Nanjing, Jiangsu Province, China. The association of DMRs with the risk and severity of sarcopenia were also investigated to identify the potential biomarkers for sarcopenia. Participants with the following conditions were excluded: (1) unable to move independently or stay in bed for a long time; (2) unable to complete the specified actions due to nervous system diseases or bone and joint diseases; (3) chronic cardiopulmonary insufficiency (unable to carry out normal daily activities; New York Heart Association heart failure was classified as grade III and IV or unable to withstand the 6‐meter walking test); (4) severe renal insufficiency (creatinine clearance rate <60 mL/min) or severe liver damage (transaminase increased more than 2 times); (5) malignant tumour; (6) mental disorders and senile dementia. The study was performed in accordance with the principles outlined in the Declaration of Helsinki^17^ and approved by the Ethics Committee of Sir Run Run Hospital, Nanjing Medical University (approval number 2019‐SR‐S041). Written informed consent was obtained from each participant. The flowchart is shown in Figure 1.

### Assessment of sarcopenia

Muscle mass was measured by the method of bioelectrical impedance analysis (BIA) by using Inbody S10 (Inbody Korea Ltd., Korea). Appendicular skeletal muscle index (ASMI) was defined as the appendicular skeletal muscle mass divided by height squared. Muscle strength was assessed by grip strength and measured using a dynamometer (CAMRY EH101, China). In standing position, the left and right handgrip strength was measured alternately three times and the maximum value was taken. Usual gait speed on a 6‐meter course was used as an objective measure of physical performance. According to the latest Asian Working Group for Sarcopenia (AWGS) 2019 criteria,^1^ patients with low muscle mass combined with low muscle strength or low physical performance were considered as moderate sarcopenia. Patients with low muscle mass combined with low muscle strength plus low physical performance were considered as severe sarcopenia. Low muscle mass was defined as an ASMI of <7.0 kg/m^2^ in men and 5.7 kg/m^2^ in women. Low handgrip strength was defined as <28 kg in men and <18 kg in women, while low physical performance was defined as a walking speed <1 m/s.

### Genome‐wide DNA methylation measurement

DNA was extracted from whole blood samples by using QIAamp® DNA Blood Mini Kit (QIAGEN, UK) following the instruction. The DNA methylation profiles of CpG sites in candidate secretary factors were determined by MethylTarget™ (Genesky Biotechnologies Inc., China), a next‐generation sequencing‐based multiple targeted CpG methylation analysis method. Primer sets were designed to flank each targeted CpG site in 100–300 nucleotide regions and are shown in Table S2. Genomic DNA was bisulfate‐converted using the EZ DNA Methylation™‐GOLD Kit (Zymo Research, CA, USA) according to the manufacturer's protocols. PCR amplicons were amplified using HotStarTaq Polymerase Kit (TAKARA, Japan) and then separated by agarose electrophoresis and purified using QIAquick Gel Extraction kit (QIAGEN, UK). After library construction, samples were sequenced on the Illumina HiSeq platform (Illumina, Inc., USA) using the paired‐end sequencing protocol according to the manufacturer's guidelines. Paired‐end reads were merged by FLASH (Fast Length Adjustment of SHort reads) and then mapped to targeted Bisulfite Genome (hg19) by blast. Mapped reads with coverage greater than 90% and identity greater than 95% were kept as effective reads and were used for following statistics. Sequencing depth for each amplicon per sample was calculated by blasting the effective reads against the targeted genome region. Reads <10‐fold were removed and overall sequencing depth for each sample were evaluated. Methylation profiles were analysed using Perl script. All analyses were operated by professional experimenters who were blind to clinical information.

### Methylation‐specific polymerase chain reaction

Bisulfate‐converted DNA was amplified by using Methylation‐specific PCR (MSP) kit (TIANGEN, China) following the manufacturer's protocol. Each reaction was performed in a total reaction volume of 20 μL, containing 2 μL MSP primer mix, 2 μL bisulfite‐treated DNA, 1.6 μL dNTPs (2.5 mM), 2 μL 10 × MSP PCR Buffer and 1 U MSP DNA polymerase (2.5 U/μL). The MSP thermal cycling program was as follows: 5 min at 95°C; 94°C for 20 s, 60°C for 30 s, and 72°C for 20 s, for a total of 35 cycles; and a final elongation step of 5 min at 72°C. Methylated and unmethylated primers were listed in Table S3. Agarose gel electrophoresis was subsequently carried out. The grey values (GV) of methylated and unmethylated bands were analysed by Image J (National Institutes of Health, USA). The following formulas were used to assess the methylation or unmethylation level: methylation level = [GVm]/[GVm + GVu] and unmethylation level = [GVu]/[GVm + GVu] (m indicated methylated and u indicated unmethylated).^18^

### Pyrosequencing

DNA methylation levels for FGF2_30 were analysed by bisulfite pyrosequencing in 50 non‐sarcopenia control subjects and 50 age‐ and sex‐matched sarcopenia patients from the above validation population. Nine samples were excluded due to poor quality control (genomic DNA < 500 ng). Finally, DNA samples of 48 non‐sarcopenia and 43 sarcopenia subjects were further bisulphite‐converted with EZ DNA methylation‐gold kits (Zymo Research Irvine, USA). The specific primers (Table S4) for PCR amplification were designed for three methylated regions using PyroMark Assay Design 2.0 software (Qiagen, Germany). While one of the methylated regions was failed to amplify, the other two methylated regions were used for pyrosequencing. The methylation level for each CpG was quantified using PyroMark Q48 (Qiagen, Germany).

### Statistical analysis

Normality of distribution was assessed using Kolmogorov–Smirnov test. Skewed data were expressed as median and quartile ranges, and comparison between two independent groups were analysed by Mann–Whitney *U* test. Pearson *χ* ^2^ test was used to compare qualitative variables represented as frequencies. Univariate analysis and multivariate logistic regression analysis were taken to determine the variables that independently contributed to sarcopenia. Odd ratios (ORs) and 95% confidence intervals (CIs) were calculated. The correlations between methylation levels and other clinical variables were calculated using Spearman correlation coefficient. The variance inflation factor (VIF) was used to quantify the severity of multicollinearity. Receiver operating characteristic (ROC) curve analysis was used to determine the optimum cut‐off levels of fibroblast growth factor 2 (FGF2)_30 methylation best predicting sarcopenia according to criteria described previously and area under the ROC curve (AUC) was measured. To analysis the power of the optimum cut‐off level for the diagnosis of sarcopenia, individuals in the validation sets were divided into training population and validation population according to a ratio of 3:7.^19^ Youden's indices (*J*) (measure of the diagnostic efficacy of the test) and Cohen's kappa coefficient (*κ*) (measure of the level of agreement between the tests) were calculated to measure the accuracy of the cut‐off value. All tests were two sided, and *P* < 0.05 was considered statistically significant. Statistical analyses were performed using PASW 25.0 (IBM SPSS, Inc., USA).

## Results

### Characteristics of the study population

In the discovery phase, we enrolled 50 patients with sarcopenia and 50 control subjects. As shown in Table S5, no difference of age and sex was observed between the two groups. The first validation population was comprised of 470 sarcopenic patients and 295 control subjects. The sarcopenic group were older (*P* < 0.001) and had more males (*P* = 0.004). The second validation population was comprised of 123 sarcopenic patients and 141 control subjects, with lower levels of BMI in the sarcopenic group BMI (*P* < 0.001). As expected, patients with sarcopenia from these two sets both had lower BMI, and lower levels of ASMI, grip strength, and gait speed (*P* < 0.001).

### DNA methylation levels

**Figure 2:**
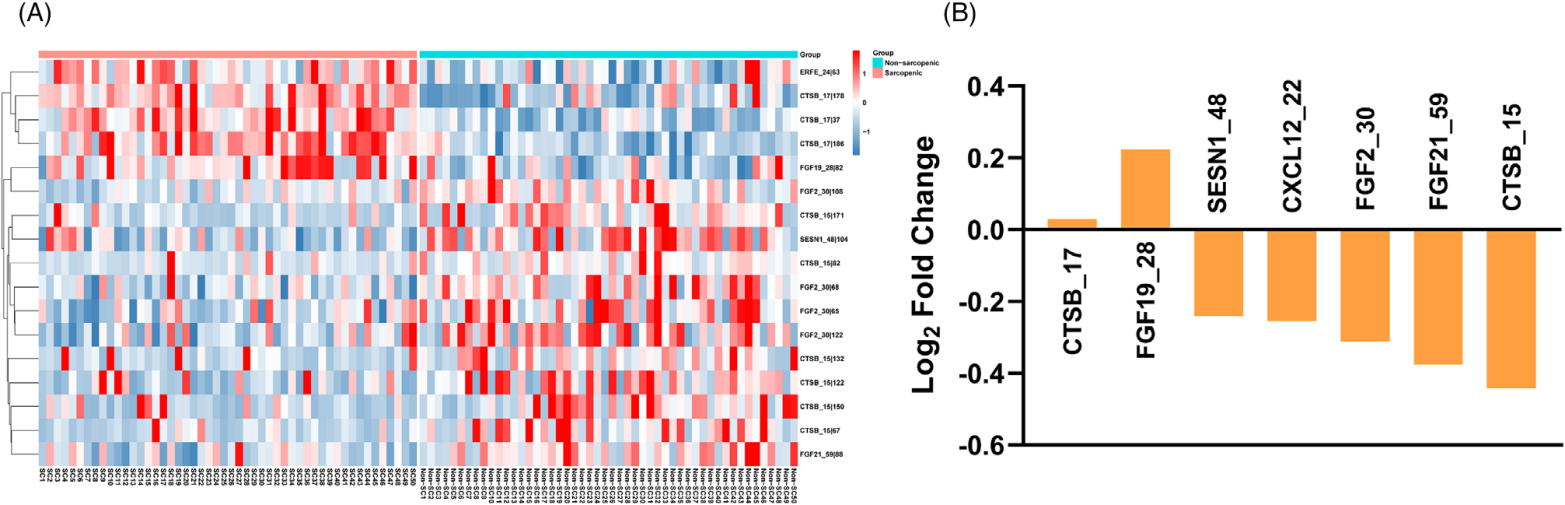
Sarcopenia‐related changes of DNA methylation in the discovery set. (A) The heatmap of dmCpG sites in sarcopenia (SC) patients and non‐sarcopenia (Non‐SC) subjects. (B) The changes of methylation levels of DMRs in patients with sarcopenia.

We first analysed the sequencing data to investigate DNA methylation levels at 1149 CpG sites of 27 skeletal muscle function‐related factors in peripheral blood samples from subjects in the discovery set. In total, 17 differentially methylated CpG (dmCpG) sites with the average *β* value >0.05 and Benjamini Hochberg false discovery rate (FDR)‐adjusted *P* value <0.05 were identified (Table S6). The dmCpG sites, including 5 hypermethylated and 12 hypomethylated dmCpG sites, were physically located within 6 genes that encoded corresponding factors and could separate most of the sarcopenia cases and controls in the clustering analysis (Figure 2A). Moreover, 7 DNA regions were significantly differentially methylated between sarcopenia patients and controls (FDR‐adjusted *P* < 0.05). These DMRs included CTSB_15, CTSB_17, CXCL12_22, FGF19_28, FGF21_59, SESN1_48, and FGF2_30, with CTSB_17 and FGF19_28 hypermethylated and the others hypomethylated in sarcopenia patients (Figure 2B and Table S6).

### Validation of differentially methylated regions

**Figure 3:**
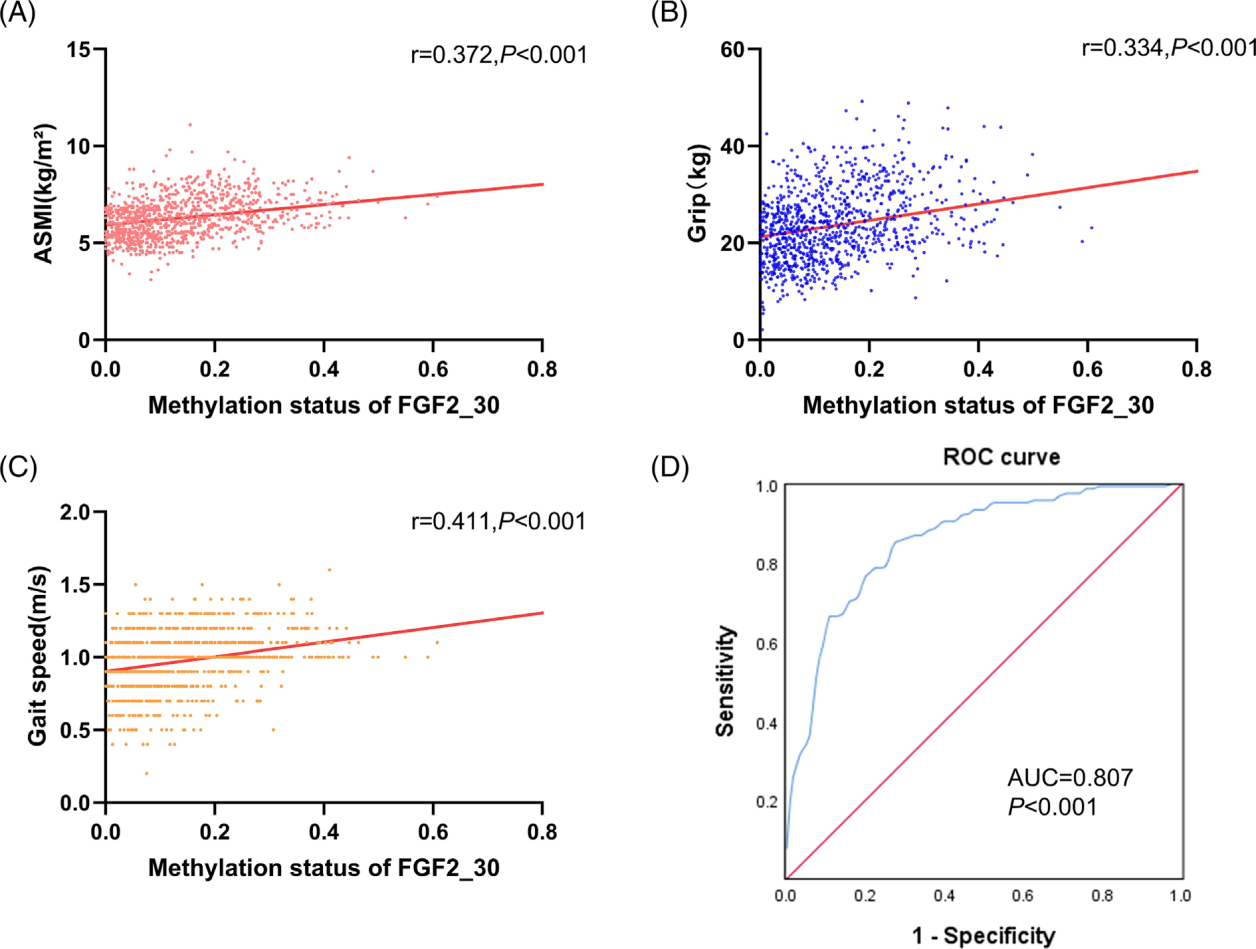
Methylation levels of FGF2_30 in the validation set. Spearman correlation coefficient was used to analyse the correlation between FGF2_30 methylation levels and ASMI (A), grip strength (B), and gait speed (C). (D) ROC curves for the diagnostic accuracy of FGF2_30 methylation for sarcopenia. ASMI, appendicular skeletal muscle mass index; AUC, area under the curve; ROC, receiver operating characteristic.

We further measured the methylation levels of 7 candidate DMRs by MSP in the first validation population. Consistently, the average methylation values were significantly lower in the sarcopenia patients at DMRs including CTSB_15, CXCL12_22, and FGF2_30. On the contrary, the methylation values were higher in the sarcopenia group at CTSB_17 and FGF19_28. However, no significant difference of SESN1_48 or FGF21_59 was observed between the two groups (Table 1 and Figure S1). Subgroup analysis based on the severity of sarcopenia showed that the methylation levels of CXCL12_22, FGF21_59, and FGF2_30 were also significantly different between patients with moderate sarcopenia and those with severe sarcopenia (Table 1). However, only the methylation level of FGF2_30 had consistent decreased trend along with the increased severity of sarcopenia, whereas the other three DMRs displayed a relationship of U‐shaped curve. We therefore further measured the methylation level of FGF2_30 in the second validation population. As expected, the methylation level of FGF2_30 was decreased as the severity of sarcopenia increased (Table 1). Further correlation analysis in the whole population demonstrated that the methylation level of FGF2_30 was positively correlated to ASMI (*r* = 0.372, *P* < 0.001), grip strength (*r* = 0.334, *P* < 0.001), and gait speed (*r* = 0.411, *P* < 0.001) (Figure 3A–C). Similar results were confirmed by pyrosequencing, showing that the methylation level of FGF2_30 was decreased with the severity of sarcopenia (Tables S7 and S8 and Figure S2A,B) and was positively associated with ASMI (*r* = 0.430, *P* < 0.001), grip strength (*r* = 0.397, *P* < 0.001) and gait speed (*r* = 0.427, *P* < 0.001) (Figure S2C–E).

**Table 1:** Methylation levels of candidate DMRs according to the sarcopenic status in the validation sets

DMRs	Non‐sarcopenia	Sarcopenia	*P*	Moderate sarcopenia	Severe sarcopenia	*P*
CTSB_15^a^	0.19 (0.13, 0.27)	0.13 (0.09, 0.19)	<0.001	0.14 (0.09, 0.19)	0.13 (0.09, 0.19)	0.125
CTSB_17 ^a^	0.54 (0.49, 0.61)	0.58 (0.53, 0.62)	<0.001	0.59 (0.52, 0.61)	0.58 (0.53, 0.62)	0.509
CXCL12_22^a^	0.73 (0.64, 0.78)	0.64 (0.54, 0.72)	<0.001	0.61 (0.48, 0.68)	0.67 (0.57, 0.76)	<0.001
FGF19_28^a^	0.29 (0.24, 0.34)	0.33 (0.26, 0.40)	<0.001	0.33 (0.25, 0.39)	0.33 (0.27, 0.41)	0.253
FGF21_59^a^	0.07 (0.04, 0.15)	0.07 (0.03, 0.12)	0.367	0.05 (0.02, 0.09)	0.08 (0.04, 0.13)	<0.001
FGF2_30^a^	0.20 (0.12, 0.27)	0.06 (0.03, 0.10)	<0.001	0.07 (0.04, 0.12)	0.05 (0.03, 0.09)	<0.001
SESN1_48^a^	0.76 (0.71, 0.81)	0.76 (0.64, 0.82)	0.414	0.76 (0.71, 0.81)	0.76 (0.45, 0.83)	0.070
FGF2_30^b^	0.19 (0.13, 0.26)	0.13 (0.09, 0.16)	<0.001	0.14 (0.09, 0.19)	0.11 (0.09, 0.15)	0.003

DMRs, differentially methylated regions.

^a^ Data from Validation set 1.

^b^ Data from Validation set 2.

### Derivation and validation of a cut‐off for FGF2_30 methylation

To analyse the potential effect of FGF2_30 methylation level on the diagnosis of sarcopenia, individuals in the validation sets were randomly divided into training population and validation population according to a ratio of 3:7.^19^ In the training population, ROC curve analysis indicated that the optimal cut‐off value of FGF2_30 methylation level that predicted sarcopenia was 0.15 (AUC = 0.807, 95% CI = 0.756–0.858, *P* < 0.001) (Figure 3D). When compared with the AWGS2019 criteria,^1^ using methylation level <0.15 as a diagnostic criteria displayed a sensitivity of 84.6% and a specificity of 70.1%, respectively, with an accuracy of 78.6% (Table 2). Similar results were observed in the validation population (Table 2 and Figure S3A), with a relatively low false positive rate of 10.4% and low false negative rate of 13.9% (Table S9).

**Table 2:** Optimal cut‐off value for the diagnosis of sarcopenia based on the methylation level of FGF2_30

Cut‐off value	Group	Sensitivity	Specificity	Accuracy	Youden's index (*J*)	Cohen's Kappa co‐efficient (*κ*)
0.15	Training population (*n* = 309)	84.6%	70.1%	78.6%	0.547	0.554
	Validation population (*n* = 720)	81.8%	67.6%	75.7%	0.494	0.499

### Association of FGF2_30 methylation with the risk of sarcopenia

Univariate and multivariate logistic regression analyses showed that lower FGF2_30 methylation level (<0.15) was significantly associated with increased risk of sarcopenia both before and after adjustment for potential confounders including age, sex, and BMI (adjusted OR = 9.223, 95% CI: 6.614–12.861, *P* < 0.001) (Table S10). Similar results were observed when using FGF2_30 methylation level as a continuous variable, showing that the risk of sarcopenia was increased along with the decreased methylation level of FGF2_30 (adjusted OR = 1.106, 95% CI: 1.086–1.126, *P* < 0.001) was decreased (Table S10).

## Discussion

To the best of our knowledge, this is the first study to explore the potential DNA methylation targeting skeletal muscle function‐related secretary factors as biomarkers for sarcopenia in community‐dwelling older adults. We found that the FGF2_30 methylation level was significantly lower in patients with sarcopenia than those non‐sarcopenic subjects and was decreased as the severity of sarcopenia increased. Moreover, older adults with lower levels of FGF2_30 methylation (<0.15) had more than 9‐fold risk of sarcopenia when compared to those with higher FGF2_30 methylation levels. Our results indicate that FGF2_30 methylation might serve as a surrogate biomarker for the screening and evaluation of sarcopenia in the older adults.

Sarcopenia has been considered as an age‐related progressive loss of muscle mass and strength that involves multistage process with the accumulation of genetic and epigenetic alterations.^5^ DNA methylation, which integrates both genetic factors and environmental exposures, has been suggested to play a prominent role in the development of sarcopenia.^5^ To date, the results of previous studies on the changes of age‐related DNA methylation in skeletal muscle have been associated with genes involved in various skeletal muscle functions, such as muscle contraction, axon guidance, calcium signalling, and so on.^20^, ^21^, ^22^, ^23^, ^24^ A recent study reported sarcopenia‐specific changes of the muscle methylome which were enriched in genes involved in myotube fusion, oxidative phosphorylation, and voltage‐gated calcium channels.^6^ Another blood‐based study on old women identified a variety of sarcopenia‐related dmCpG sites involved in many muscle‐related physiological processes, such as muscle differentiation, muscle function, and energy metabolism.^7^ Intriguingly, we here also identified a panel of sarcopenia‐associated DMRs targeting skeletal muscle function‐related secretary factors as potential biomarkers for sarcopenia in community‐dwelling older adults. These results are consistent with the endocrine characteristic of skeletal muscle, indicating that sarcopenia‐related changes in DNA methylation are not only restricted to the skeletal muscle itself, but may also occurred in various tissues. Therefore, blood‐based DNA methylation examination might be a potential promising non‐invasive diagnostic tool for sarcopenia.

An interesting finding of our study was the U‐shaped association of the methylation levels of CXCL12_22 and FGF21_59 with the severity of sarcopenia. CXCL12, also known as stromal cell‐derived factor 1, has been implicated in the regulation of skeletal muscle development and has protective effect against muscle damage through promoting muscle regeneration.^25^, ^26^ Similarly, as a pleiotropic hormone, FGF21 has also been demonstrated to attenuate muscle atrophy and promote muscle regeneration under different stimulus that lead to skeletal muscle damage.^27^, ^28^ Under the condition of gene promoters, hypomethylated status is mainly associated with activated gene expression, while hypermethylated status is usually associated with suppressed gene expression.^29^ Therefore, we hypothesized that the observed hypomethylation of CXCL12_22 and FGF21_59 in moderate sarcopenia might indicate a compensatory increased secretion of CXCL12 and FGF21, whereas sustained secretion may result in decreased levels of these two factors, reflecting as hypermethylation of CXCL12_22 and FGF21_59 in patients with severe sarcopenia. Nevertheless, it is still arbitrary to assign a favourable or harmful role to the altered methylation status of CXCL12 and FGF21 for skeletal muscle health without functional study.

Our results demonstrated that DMR in FGF2 was correlated with the risk and severity of sarcopenia. When compared to the AWGS2019 criteria,^1^ using <0.15 as an optimal cut‐off value of FGF2_30 methylation level displayed relatively high sensitivity and specificity as well as relatively low false negative rate in the diagnosis of sarcopenia both in the training population and validation population. Moreover, we observed similar ability of prediction of sarcopenia between using combined 7‐DMRs and using single FGF2_30 DMR (Figure S3B), indicating a potential application of examining FGF2_30 methylation level for the screening of sarcopenia. DNA methylation is usually associated with transcriptional silencing.^29^ Considering that the position of FGF2_30 segment is located in the promoter of FGF2 (Figure S4), the observed lower FGF2_30 methylation level in patients with sarcopenia may therefore indicate a potential role of elevated FGF2 in the pathogenesis of sarcopenia. FGF2, which is also known as basic fibroblast growth factor (bFGF), has been identified to play important roles in the skeletal development and muscle growth.^30^ Under physiological conditions, FGF2 promotes regenerative myogenesis by stimulating the proliferation of satellite cells.^31^ Aging increases the expression of skeletal muscle FGF2, leading to continuous depletion of satellite cells pool and ultimately loss of muscle.^32^ Neutralizing antibody to FGF2 was shown to attenuate skeletal muscle injury.^33^ However, another study found that FGF2 expression was decreased in skeletal muscle of old mice compared with young mice.^34^ Furthermore, knockout of FGF2 exacerbated age‐related inflammatory and fibrotic changes in skeletal muscle in old mice.^34^ These paradoxical results might be explained by the declined FGF2 sensitivity of aged satellite cells during their proliferation and self‐renewal.^35^ Rozo et al. reported that activating β1‐integrin signalling could restore FGF2 sensitivity in aged satellite cells and improve regeneration after muscle injury.^36^ Another possible explanation might be associated with the different functions of FGF2 in different muscle fibre types.^37^ Further in‐depth studies will be needed to elucidate the precise mechanism governing the pathological effect of FGF2 on the development of sarcopenia.

DNA methylation alterations can either be inherited and persist for years or be *de novo* modified under various types of stresses.^29^ The maintenance of DNA methylation is carried out by DNA methyltransferase 1 (DNMT1) while the *de novo* methylation is catalysed by DNMT3a and DNMT3b.^29^ During aging, the genome is generally hypomethylated mainly due to the decreased expression of DNMT1.^38^ By contrast, the expression of DNMT3a and DNMT3b increases with age, resulting in increased *de novo* methylation of CpG islands in mammalian cells.^38^ Moreover, DNA methylation can be removed by ten‐eleven translocation (TET) enzymes, the changes of which may also impact the phenotype of aging.^39^ Further loss‐ and gain‐of‐function experiments of DNMTs or TETs will help to explore the exact mechanisms by which causes the hypomethylation of FGF2 in sarcopenia.

## Study limitations

Firstly, although our study population was consistent of discovery and validation sets from both rural and urban areas, it was still hard to exclude the possibility of selection and causality bias as a result of the cross‐sectional nature. Further prospective cohort studies will be needed to confirm our findings. Secondly, although our results suggest lower FGF2_30 methylation might be a potential biomarker for sarcopenia, we did not examine the serum level of FGF2 in patients with sarcopenia. Further studies are needed to investigate the correlation between FGF2_30 methylation status and serum FGF2 level in sarcopenia patients.

## Conclusions

In summary, our findings demonstrate that lower FGF2_30 methylation is correlated with the risk and severity of sarcopenia in the older adults. As an objective biomarker, addition of FGF2_30 methylation examination to the traditional diagnostic methods may help to facilitate the early screening of patients with sarcopenia. Further studies are needed to confirm the results of our study and explore the underlying mechanism for the association of FGF2 DNA methylation with sarcopenia.

## Conflict of interest

The authors declare no conflict of interest.

## Supporting information


**Figure S1.** The methylation levels of DMRs were analysed by MSP with methylated and unmethylated‐specific primers. The methylation levels of CTSB_15 (A), CTSB_17 (B), CXCL12_22 (C), FGF19_28 (D), FGF21_59 (E), FGF2_30 (F), and SESN1_48 (G) in different subgroups according to the status of sarcopenia. Upper: the representative agarose gel pictures of methylation and unmethylation bands. Lower: the relative methylation levels. M: methylation bands; U: unmethylation bands. **, *P* < 0.01; ***, *P* < 0.001; NS, no significance.

**Figure S2.** Methylation levels of FGF2_30 by pyrosequencing. (**A**) The levels of FGF2_30 methylation in subjects with and without sarcopenia. (**B**) The levels of FGF2_30 methylation in subjects with different stages of sarcopenia. Spearman correlation coefficient was used to analyse the correlation between FGF2_30 methylation levels and ASMI (**C**), grip strength (**D**), and gait speed (**E**). (**D**) ROC curves for the diagnostic accuracy of FGF2_30 methylation for sarcopenia. ROC, receiver operating characteristic; AUC, aera under the curve, ASMI, appendicular skeletal muscle mass index. *, *P* < 0.05; ***, *P* < 0.001.

**Figure S3.** (**A**) ROC curves for the diagnostic accuracy of FGF2_30 methylation for sarcopenia in the validation population. (**B**) ROC curves for the diagnostic accuracy between using combined 7‐DMRs and using single FGF2_30 DMR. ROC, receiver operating characteristic; AUC, aera under the curve

**Figure S4.** The scheme of the position of FGF2_30 segement.

**Table S1.** The detailed information of selected myokines in the present study.

**Table S2.** Supporting Information

**Table S3.** Primers used for methylation‐specific PCR analysis (MSP) and amplification conditions.

**Table S4.** The primers of FGF2_30 for PCR amplification by pyrosequencing.

**Table S5.** Characteristics of subjects in the discovery and validation set.

**Table S6.** Supporting Information

**Table S7.** Characteristics of subjects in the pyrosequencing.

**Table S8.** Supporting Information

**Table S9.** Concordance between the two sarcopenia evaluation methods in the validation population.

**Table S10.** Associations of methylation levels of FGF2_30 with the Risk of Sarcopenia.
