# Supplementary material for: DNA methylation of skeletal muscle function‐related secretary factors identifies FGF2 as a potential biomarker for sarcopenia
Source: J Cachexia Sarcopenia Muscle. 2024 Apr 20;15(3):1209–17. doi: 10.1002/jcsm.13472 (PMC11154778; doi:10.1002/jcsm.13472)
Supplement: Supplementary file 1 — Figure S1. The methylation levels of DMRs were analysed by MSP with methylated and unmethylated‐specific primers. The methylation levels of CTSB_15 (A), CTSB_17 (B), CXCL12_22 (C), FGF19_28 (D), FGF21_59 (E), FGF2_30 (F), and SESN1_48 (G) in different subgroups according to the status of sarcopenia. Upper: the representative agarose gel pictures of methylation and unmethylation bands. Lower: the relative methylation levels. M: methylation bands; U: unmethylation bands. **, P < 0.01; ***, P < 0.001; NS, no significance. [file JCSM-15-1209-s004.docx]

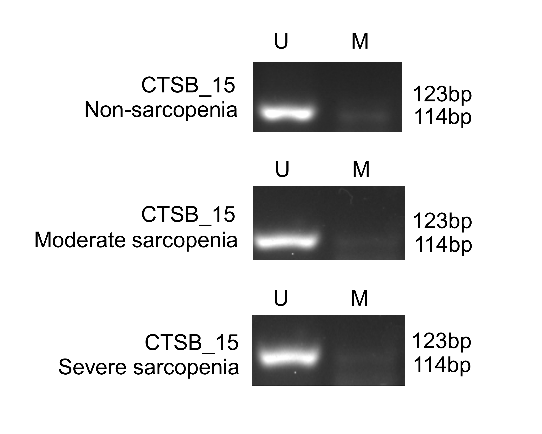

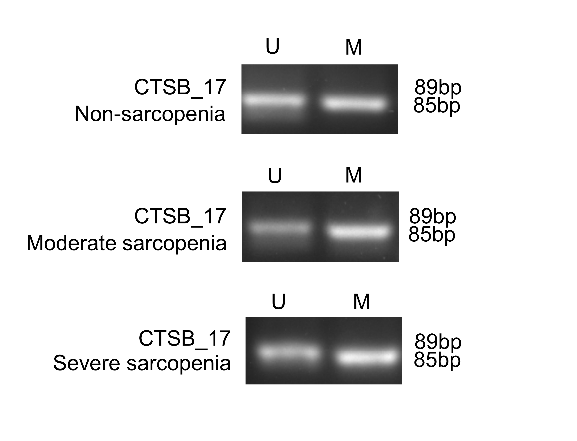


B

A


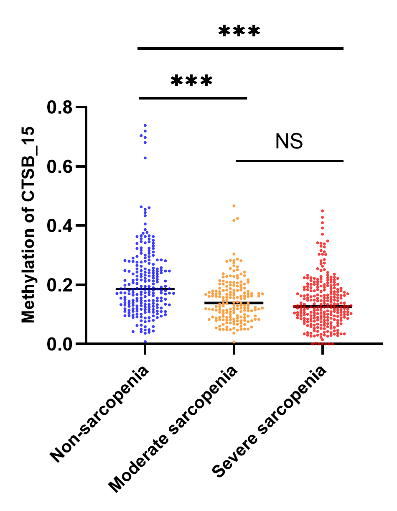

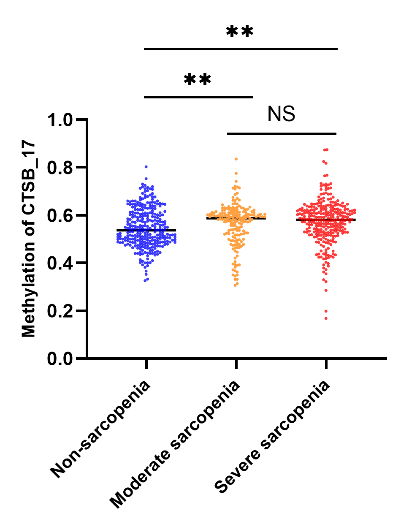


D

C


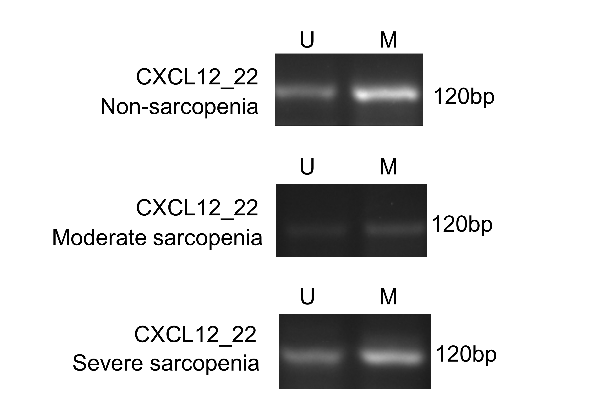

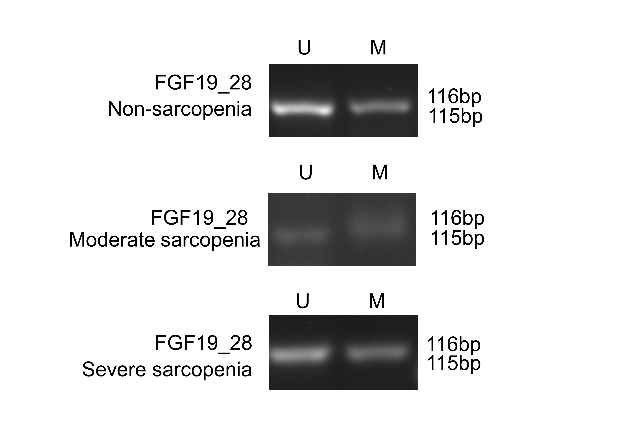


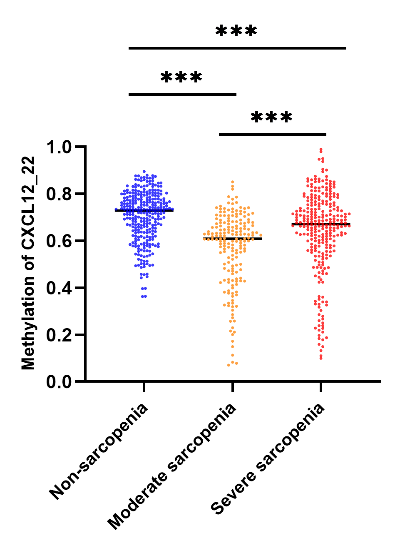

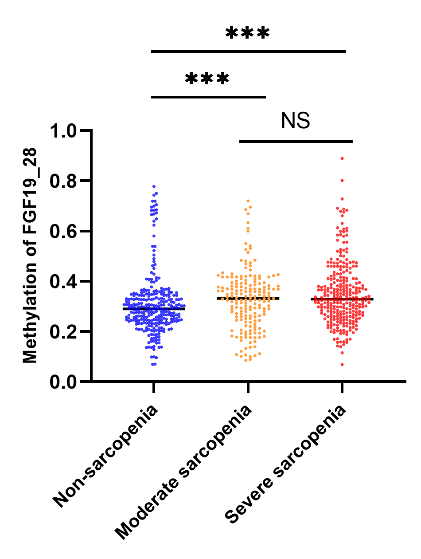


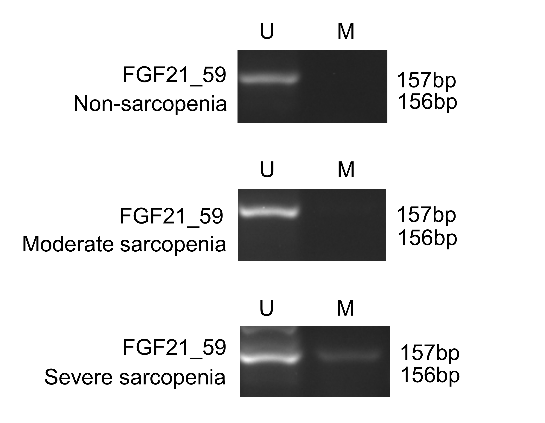

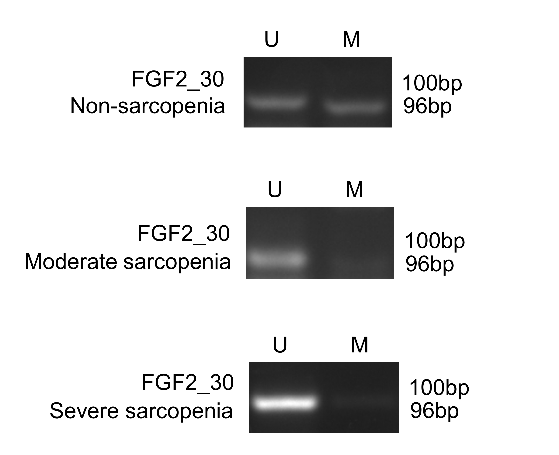


E

F


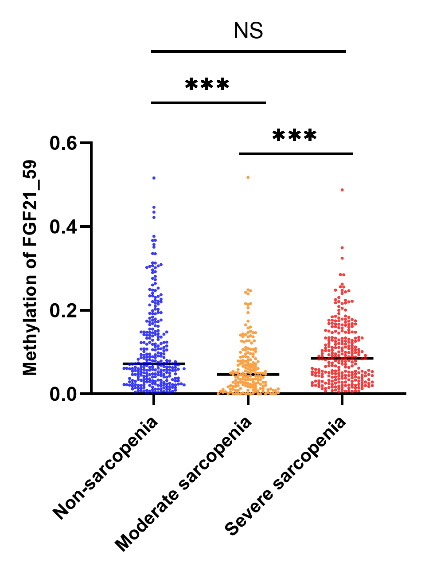

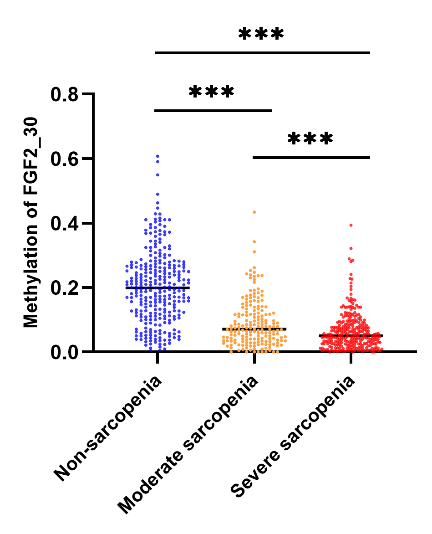


G


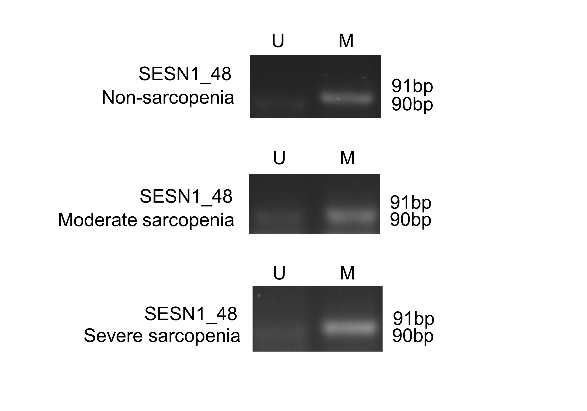


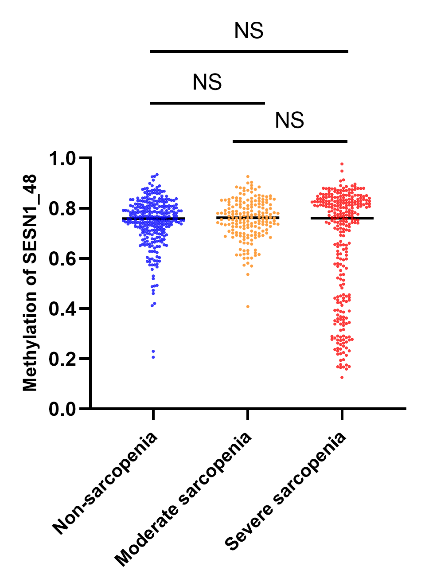


**Figure S1**. The methylation levels of DMRs were analyzed by MSP with methylated and unmethylated-specific primers. The methylation levels of CTSB_15 (A), CTSB_17 (B), CXCL12_22 (C), FGF19_28 (D), FGF21_59 (E), FGF2_30 (F), and SESN1_48 (G) in different subgroups according to the status of sarcopenia. Upper: the representative agarose gel pictures of methylation and unmethylation bands. Lower: the relative methylation levels. M: methylation bands; U: unmethylation bands. **, *P*<0.01; ***, *P*<0.001; NS, no significance.
