# Supplementary material for: DNA methylation of skeletal muscle function‐related secretary factors identifies FGF2 as a potential biomarker for sarcopenia
Source: J Cachexia Sarcopenia Muscle. 2024 Apr 20;15(3):1209–17. doi: 10.1002/jcsm.13472 (PMC11154778; doi:10.1002/jcsm.13472)
Supplement: Supplementary file 2 — Figure S2. Methylation levels of FGF2_30 by pyrosequencing. (A) The levels of FGF2_30 methylation in subjects with and without sarcopenia. (B) The levels of FGF2_30 methylation in subjects with different stages of sarcopenia. Spearman correlation coefficient was used to analyse the correlation between FGF2_30 methylation levels and ASMI (C), grip strength (D), and gait speed (E). (D) ROC curves for the diagnostic accuracy of FGF2_30 methylation for sarcopenia. ROC, receiver operating characteristic; AUC, aera under the curve, ASMI, appendicular skeletal muscle mass index. *, P < 0.05; ***, P < 0.001. [file JCSM-15-1209-s012.docx]

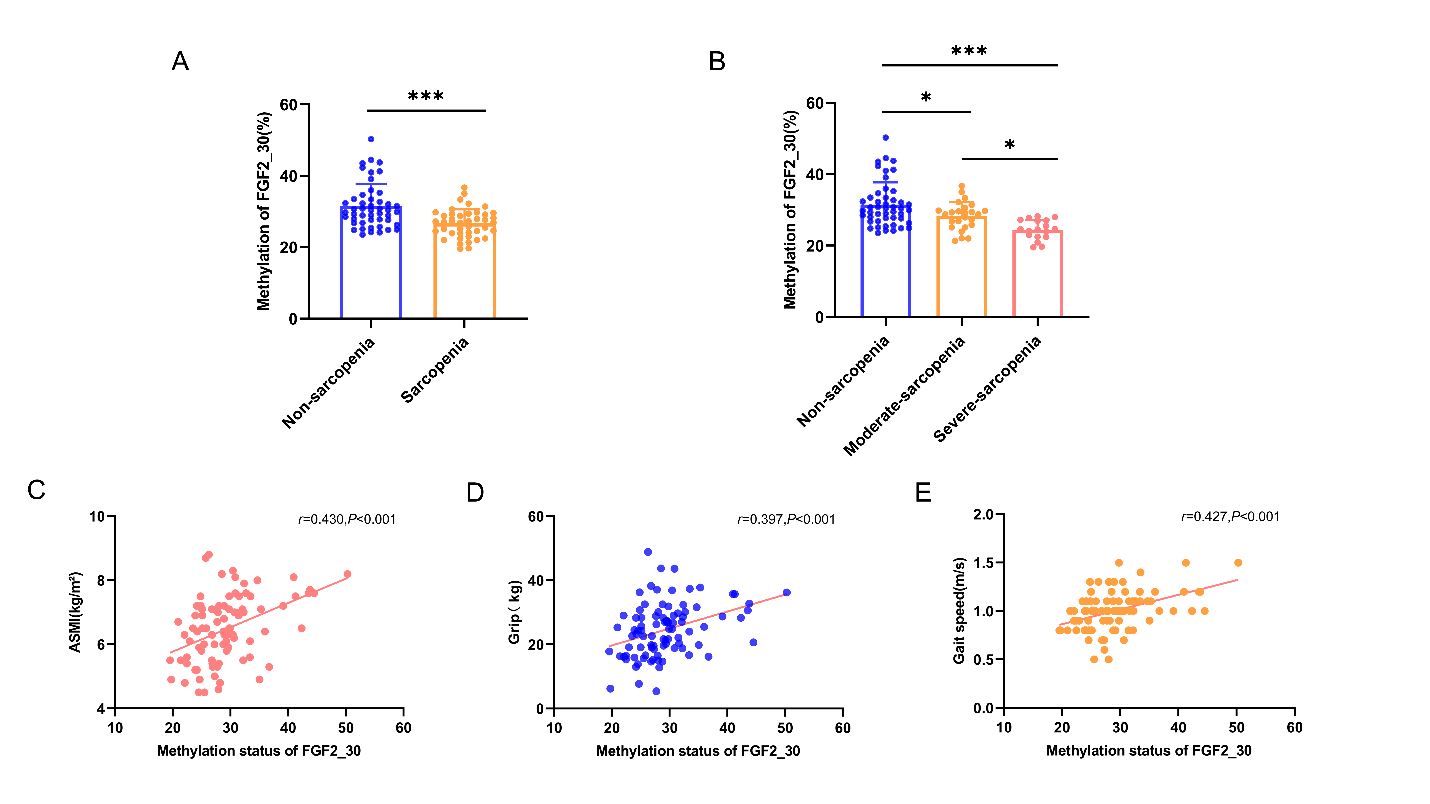


**Figure S2.** Methylation levels of FGF2_30 by pyrosequencing. (**A**) The levels of FGF2_30 methylation in subjects with and without sarcopenia. (**B**) The levels of FGF2_30 methylation in subjects with different stages of sarcopenia. Spearman correlation coefficient was used to analyze the correlation between FGF2_30 methylation levels and ASMI (**C**), grip strength (**D**), and gait speed (**E**). (**D**) ROC curves for the diagnostic accuracy of FGF2_30 methylation for sarcopenia. ROC, receiver operating characteristic; AUC, aera under the curve, ASMI, appendicular skeletal muscle mass index. *, *P*<0.05; ***, *P*<0.001.
