# Supplementary material for: DNA methylation of skeletal muscle function‐related secretary factors identifies FGF2 as a potential biomarker for sarcopenia
Source: J Cachexia Sarcopenia Muscle. 2024 Apr 20;15(3):1209–17. doi: 10.1002/jcsm.13472 (PMC11154778; doi:10.1002/jcsm.13472)
Supplement: Supplementary file 3 — Figure S3. (A) ROC curves for the diagnostic accuracy of FGF2_30 methylation for sarcopenia in the validation population. (B) ROC curves for the diagnostic accuracy between using combined 7‐DMRs and using single FGF2_30 DMR. ROC, receiver operating characteristic; AUC, aera under the curve [file JCSM-15-1209-s007.docx]

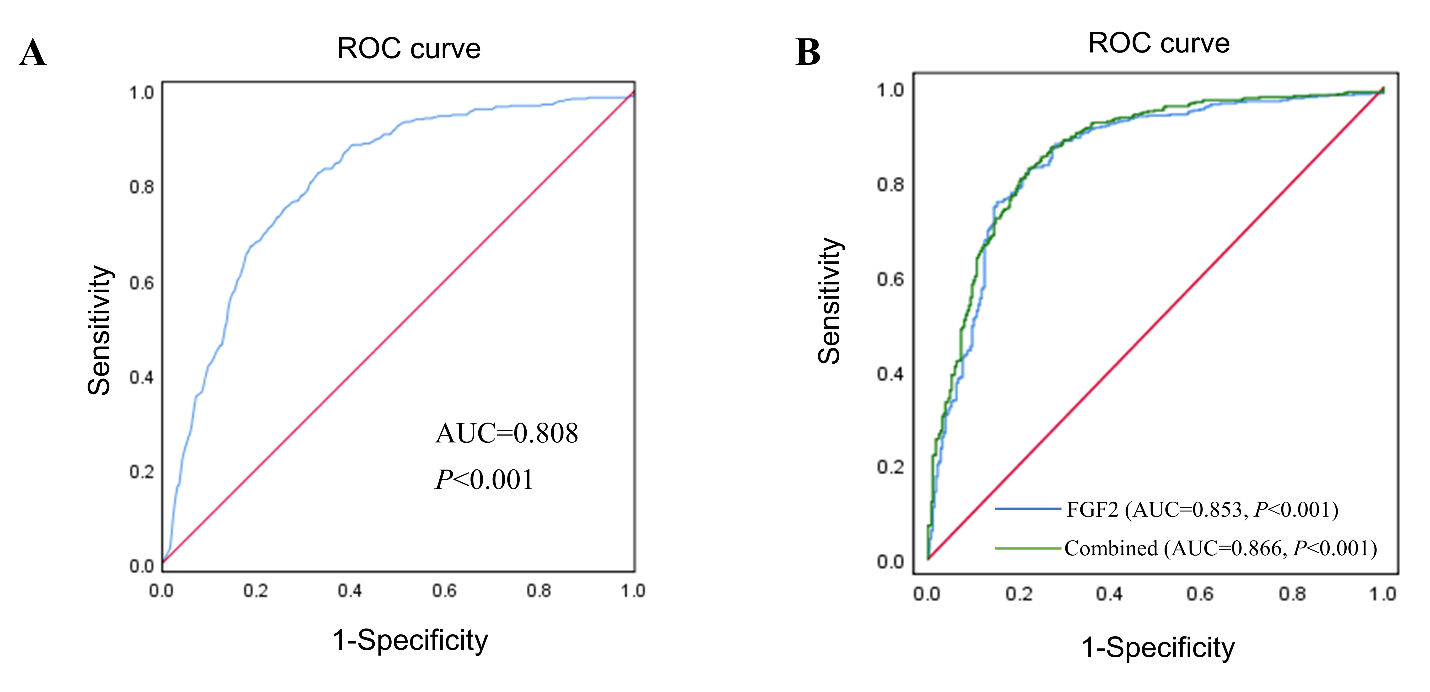


**Figure S3.** (**A**) ROC curves for the diagnostic accuracy of FGF2_30 methylation for sarcopenia in the validation population. (**B**) ROC curves for the diagnostic accuracy between using combined 7-DMRs and using single FGF2_30 DMR. ROC, receiver operating characteristic; AUC, aera under the curve
