# Supplementary figures and images for: DNA methylation of skeletal muscle function‐related secretary factors identifies FGF2 as a potential biomarker for sarcopenia
Source: J Cachexia Sarcopenia Muscle. 2024 Apr 20;15(3):1209–17. doi: 10.1002/jcsm.13472 (PMC11154778; doi:10.1002/jcsm.13472)

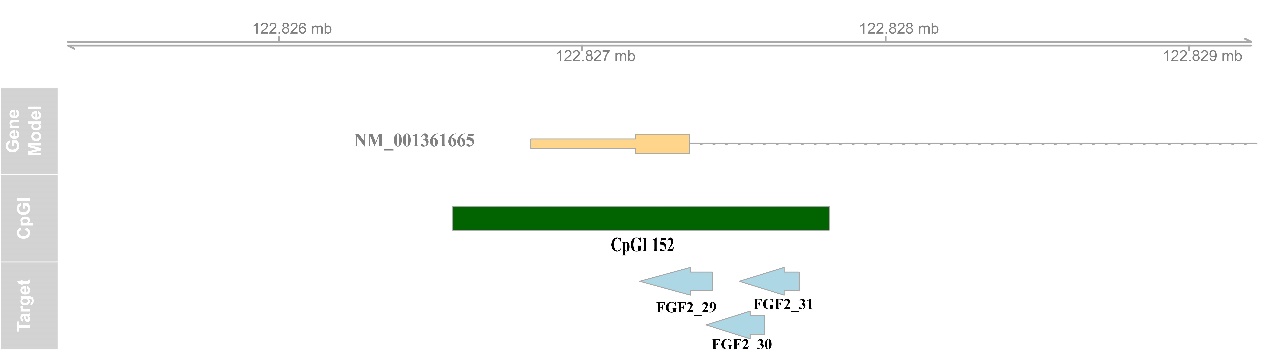


**Figure S4.** The scheme of the position of FGF2_30 segement.

Supplement: Supplementary file 4 — Figure S4. The scheme of the position of FGF2_30 segement. [file JCSM-15-1209-s006.docx]
