# Supplementary material for: DNA methylation of skeletal muscle function‐related secretary factors identifies FGF2 as a potential biomarker for sarcopenia
Source: J Cachexia Sarcopenia Muscle. 2024 Apr 20;15(3):1209–17. doi: 10.1002/jcsm.13472 (PMC11154778; doi:10.1002/jcsm.13472)
Supplement: Supplementary file 5 — Table S1. The detailed information of selected myokines in the present study. [file JCSM-15-1209-s011.docx]

**Table S1.** The detailed information of selected myokines in the present study.

| **Gene name** | | **Gene symbol** | **Chromosome** | **Biological function** | **Circulating level in certain condition** |
| --- | --- | --- | --- | --- | --- |
| Angiopoietin-like 4 | | ANGPTL4 | Chr 19 | Increase the maximum mitochondrial oxidative capacity in skeletal muscle [1] | Increased serum levels after acute exercise[2] |
| Apelin | | APLN | Chr X | Regulate skeletal muscle plasticity and metabolism [3] | Decreased serum levels in osteoporosis [4] |
| Brain-derived neurotrophic factor | | BDNF | Chr 11 | Maintain muscle function and plasticity [5] | Increased serum levels after acute exercise[6] |
| Bone morphogenetic protein 7 | | BMP7 | Chr 20 | Induce myotube formation [7] | Not determined |
| Cathepsin B | | CTSB | Chr 8 | Degrade cellular skeletal muscle proteins [8] | Increased serum levels after aerobic exercise training [9] |
| Stromal derived factor-1 | | SDF1 | Chr 10 | Promote skeletal muscle regeneration [7] | Increased serum levels in low bone mineral density [10] |
| Interleukin 8 | | IL8 | Chr 4 | Induce angiogenesis by promoting angiogenic responses in endothelial cells [11] | Increased serum levels in sarcopenia [12] |
| Erythropoietin | | EPO | Chr 7 | Promote myoblast differentiation and survival [13] | Not determined |
| Erythroferrone | | ERFE | Chr 2 | Enhance glucose uptake and fatty acid oxidation in skeletal muscle [14] | Decreased serum levels after exercise [14] |
| Fibroblast growth factor 19 | | FGF19 | Chr 11 | Induce skeletal muscle hypertrophy and blocked muscle atrophy [15] | Decreased serum levels in sarcopenia [16] |
| Fibroblast growth factor 2 | | FGF2 | Chr 4 | Regulate muscle regeneration and maintenance by increased proliferation of satellite cells [17] | Not determined |
| Fibroblast growth factor 21 | FGF21 | | Chr 19 | Enhance glucose uptake and insulin sensitization in skeletal muscle [18] | Increased serum levels in sarcopenia [16] |
| Fibronectin type III domain-containing protein 5 | | FNDC5 | Chr 1 | Protect against muscle wasting [19] | Increased serum levels in sarcopenia [20] |
| Follistatin | | FST | Chr 5 | Inhibit myostatin-mediated muscle wasting [21] | Increased serum levels in older women with lower muscle power [22] |
| Follistatin-like protein 1 | | FSTL1 | Chr 3 | Increase glucose uptake in skeletal muscle [23] | Increased serum levels after acute exercise [24] |
| Growth differentiating factor 15 | | GDF15 | Chr 19 | Induce muscle atrophy [25] | Increased serum levels in sarcopenia [26] |
| Interleukin 15 | | IL15 | Chr 4 | Decrease muscle protein degradation [27] | Increased serum levels after resistance exercise [28] |
| Interleukin 6 | | IL6 | Chr 7 | A double‐edged sword: stimulate the proliferative capacity of muscle stem cells and promote muscle atrophy [29] | Increased serum levels in sarcopenia [30] |
| Interleukin 7 | | IL7 | Chr 8 | Act on satellite cells to inhibit the development of muscle fiber phenotype [31] | Increased serum levels in cancer cachexia [32] |
| Leukemia inhibitory factor | | LIF | Chr 22 | Induce proliferation of satellite cells [33] | Unchanged after resistance training [34] |
| Meteorin‐like | | METRNL | Chr 17 | Promote muscle regeneration [35] | Not determined |
| Retinol-binding protein 4 | | RBP4 | Chr 10 | Inhibit the glucose uptake of skeletal muscle [36] | Increased serum levels in sarcopenia [36] |
| Sestrin | | SESN1 | Chr 6 | Prevent muscle atrophy [7] | Decreased serum levels of SESN1 and SESN2 in frail elderly [37] |
|  |  | SESN2 | Chr 1 |  |  |
|  |  | SESN3 | Chr 11 |  |  |
| Secreted protein acidic and rich in cysteine | | SPARC | Chr 5 | Inhibit myoblast differentiation [38] | Increased serum levels after acute exercise [39] |
| Vascular endothelial growth factor A | | VEGFA | Chr 6 | Promote growth and survival of skeletal muscle [7] | Increased serum levels in colorectal cancer with low skeletal muscle area [40] |

**References:**

1. Chang H, Kwon O, Shin MS, Kang GM, Leem YH, Lee CH, et al. Role of Angptl4/Fiaf in exercise-induced skeletal muscle AMPK activation. Journal of applied physiology (Bethesda, Md : 1985). 2018;125:715-22. doi:10.1152/japplphysiol.00984.2016

2. Li G, Zhang H, Ryan AS. Skeletal Muscle Angiopoietin-Like Protein 4 and Glucose Metabolism in Older Adults after Exercise and Weight Loss. Metabolites. 2020;10:doi:10.3390/metabo10090354

3. Lee U, Stuelsatz P, Karaz S, McKellar DW, Russeil J, Deak M, et al. A Tead1-Apelin axis directs paracrine communication from myogenic to endothelial cells in skeletal muscle. iScience. 2022;25:104589. doi:10.1016/j.isci.2022.104589

4. Liu S, Wang W, Yin L, Zhu Y. Influence of Apelin-13 on osteoporosis in Type-2 diabetes mellitus: A clinical study. Pakistan journal of medical sciences. 2018;34:159-63. doi:10.12669/pjms.341.14135

5. Rentería I, García-Suárez PC, Fry AC, Moncada-Jiménez J, Machado-Parra JP, Antunes BM, et al. The Molecular Effects of BDNF Synthesis on Skeletal Muscle: A Mini-Review. Frontiers in physiology. 2022;13:934714. doi:10.3389/fphys.2022.934714

6. Matthews VB, Aström MB, Chan MH, Bruce CR, Krabbe KS, Prelovsek O, et al. Brain-derived neurotrophic factor is produced by skeletal muscle cells in response to contraction and enhances fat oxidation via activation of AMP-activated protein kinase. Diabetologia. 2009;52:1409-18. doi:10.1007/s00125-009-1364-1

7. Kwon JH, Moon KM, Min KW. Exercise-Induced Myokines can Explain the Importance of Physical Activity in the Elderly: An Overview. Healthcare (Basel, Switzerland). 2020;8:doi:10.3390/healthcare8040378

8. Bosutti A, Toigo G, Ciocchi B, Situlin R, Guarnieri G, Biolo G. Regulation of muscle cathepsin B proteolytic activity in protein-depleted patients with chronic diseases. Clinical nutrition (Edinburgh, Scotland). 2002;21:373-8. doi:10.1054/clnu.2002.0557

9. Moon HY, Becke A, Berron D, Becker B, Sah N, Benoni G, et al. Running-Induced Systemic Cathepsin B Secretion Is Associated with Memory Function. Cell metabolism. 2016;24:332-40. doi:10.1016/j.cmet.2016.05.025

10. Carbone LD, Bůžková P, Fink HA, Robbins JA, Bethel M, Hamrick MW, et al. Association of Plasma SDF-1 with Bone Mineral Density, Body Composition, and Hip Fractures in Older Adults: The Cardiovascular Health Study. Calcified tissue international. 2017;100:599-608. doi:10.1007/s00223-017-0245-8

11. Yoon JH, Kim J, Song P, Lee TG, Suh PG, Ryu SH. Secretomics for skeletal muscle cells: a discovery of novel regulators? Advances in biological regulation. 2012;52:340-50. doi:10.1016/j.jbior.2012.03.001

12. Hou YC, Wang CJ, Chao YJ, Chen HY, Wang HC, Tung HL, et al. Elevated Serum Interleukin-8 Level Correlates with Cancer-Related Cachexia and Sarcopenia: An Indicator for Pancreatic Cancer Outcomes. Journal of clinical medicine. 2018;7:doi:10.3390/jcm7120502

13. Lamon S, Russell AP. The role and regulation of erythropoietin (EPO) and its receptor in skeletal muscle: how much do we really know? Frontiers in physiology. 2013;4:176. doi:10.3389/fphys.2013.00176

14. Raschke S, Eckel J. Adipo-myokines: two sides of the same coin--mediators of inflammation and mediators of exercise. Mediators of inflammation. 2013;2013:320724. doi:10.1155/2013/320724

15. Benoit B, Meugnier E, Castelli M, Chanon S, Vieille-Marchiset A, Durand C, et al. Fibroblast growth factor 19 regulates skeletal muscle mass and ameliorates muscle wasting in mice. Nature medicine. 2017;23:990-6. doi:10.1038/nm.4363

16. Bag Soytas R, Suzan V, Arman P, Emiroglu Gedik T, Unal D, Cengiz M, et al. Association of FGF-19 and FGF-21 levels with primary sarcopenia. Geriatrics & gerontology international. 2021;21:959-62. doi:10.1111/ggi.14263

17. Kim JS, Yoon DH, Kim HJ, Choi MJ, Song W. Resistance exercise reduced the expression of fibroblast growth factor-2 in skeletal muscle of aged mice. Integrative medicine research. 2016;5:230-5. doi:10.1016/j.imr.2016.05.001

18. Sun H, Sherrier M, Li H. Skeletal Muscle and Bone - Emerging Targets of Fibroblast Growth Factor-21. Frontiers in physiology. 2021;12:625287. doi:10.3389/fphys.2021.625287

19. Colaianni G, Mongelli T, Colucci S, Cinti S, Grano M. Crosstalk Between Muscle and Bone Via the Muscle-Myokine Irisin. Current osteoporosis reports. 2016;14:132-7. doi:10.1007/s11914-016-0313-4

20. Chang JS, Kim TH, Nguyen TT, Park KS, Kim N, Kong ID. Circulating irisin levels as a predictive biomarker for sarcopenia: A cross-sectional community-based study. Geriatrics & gerontology international. 2017;17:2266-73. doi:10.1111/ggi.13030

21. Kalinkovich A, Livshits G. Sarcopenia--The search for emerging biomarkers. Ageing research reviews. 2015;22:58-71. doi:10.1016/j.arr.2015.05.001

22. Fife E, Kostka J, Kroc Ł, Guligowska A, Pigłowska M, Sołtysik B, et al. Relationship of muscle function to circulating myostatin, follistatin and GDF11 in older women and men. BMC geriatrics. 2018;18:200. doi:10.1186/s12877-018-0888-y

23. Lee HJ, Lee JO, Lee YW, Kim SA, Park SH, Kim HS. Kalirin, a GEF for Rac1, plays an important role in FSTL-1-mediated glucose uptake in skeletal muscle cells. Cellular signalling. 2017;29:150-7. doi:10.1016/j.cellsig.2016.10.013

24. Görgens SW, Raschke S, Holven KB, Jensen J, Eckardt K, Eckel J. Regulation of follistatin-like protein 1 expression and secretion in primary human skeletal muscle cells. Archives of physiology and biochemistry. 2013;119:75-80. doi:10.3109/13813455.2013.768270

25. Zhang W, Sun W, Gu X, Miao C, Feng L, Shen Q, et al. GDF-15 in tumor-derived exosomes promotes muscle atrophy via Bcl-2/caspase-3 pathway. Cell death discovery. 2022;8:162. doi:10.1038/s41420-022-00972-z

26. Kim M, Walston JD, Won CW. Associations Between Elevated Growth Differentiation Factor-15 and Sarcopenia Among Community-dwelling Older Adults. The journals of gerontology Series A, Biological sciences and medical sciences. 2022;77:770-80. doi:10.1093/gerona/glab201

27. Li F, Li Y, Duan Y, Hu CA, Tang Y, Yin Y. Myokines and adipokines: Involvement in the crosstalk between skeletal muscle and adipose tissue. Cytokine & growth factor reviews. 2017;33:73-82. doi:10.1016/j.cytogfr.2016.10.003

28. Quinn LS, Anderson BG, Strait-Bodey L, Stroud AM, Argilés JM. Oversecretion of interleukin-15 from skeletal muscle reduces adiposity. American journal of physiology Endocrinology and metabolism. 2009;296:E191-202. doi:10.1152/ajpendo.90506.2008

29. Muñoz-Cánoves P, Scheele C, Pedersen BK, Serrano AL. Interleukin-6 myokine signaling in skeletal muscle: a double-edged sword? The FEBS journal. 2013;280:4131-48. doi:10.1111/febs.12338

30. Picca A, Coelho-Junior HJ, Calvani R, Marzetti E, Vetrano DL. Biomarkers shared by frailty and sarcopenia in older adults: A systematic review and meta-analysis. Ageing research reviews. 2022;73:101530. doi:10.1016/j.arr.2021.101530

31. Haugen F, Norheim F, Lian H, Wensaas AJ, Dueland S, Berg O, et al. IL-7 is expressed and secreted by human skeletal muscle cells. American journal of physiology Cell physiology. 2010;298:C807-16. doi:10.1152/ajpcell.00094.2009

32. Costa RGF, Caro PL, de Matos-Neto EM, Lima J, Radloff K, Alves MJ, et al. Cancer cachexia induces morphological and inflammatory changes in the intestinal mucosa. Journal of cachexia, sarcopenia and muscle. 2019;10:1116-27. doi:10.1002/jcsm.12449

33. Barbalho SM, Prado Neto EV, De Alvares Goulart R, Bechara MD, Baisi Chagas EF, Audi M, et al. Myokines: a descriptive review. The Journal of sports medicine and physical fitness. 2020;60:1583-90. doi:10.23736/s0022-4707.20.10884-3

34. Leuchtmann AB, Adak V, Dilbaz S, Handschin C. The Role of the Skeletal Muscle Secretome in Mediating Endurance and Resistance Training Adaptations. Frontiers in physiology. 2021;12:709807. doi:10.3389/fphys.2021.709807

35. Alizadeh H. Meteorin-like protein (Metrnl): A metabolic syndrome biomarker and an exercise mediator. Cytokine. 2022;157:155952. doi:10.1016/j.cyto.2022.155952

36. Chang CL, Li YR, Wang ZY, Li ML, Jia KY, Sun HX, et al. Serum Retinol Binding Protein 4 as a Potential Biomarker for Sarcopenia in Older Adults. The journals of gerontology Series A, Biological sciences and medical sciences. 2022;doi:10.1093/gerona/glac151

37. Rai N, Venugopalan G, Pradhan R, Ambastha A, Upadhyay AD, Dwivedi S, et al. Exploration of Novel Anti-Oxidant Protein Sestrin in Frailty Syndrome in Elderly. Aging and disease. 2018;9:220-7. doi:10.14336/ad.2017.0423

38. Petersson SJ, Jørgensen LH, Andersen DC, Nørgaard RC, Jensen CH, Schrøder HD. SPARC is up-regulated during skeletal muscle regeneration and inhibits myoblast differentiation. Histology and histopathology. 2013;28:1451-60. doi:10.14670/hh-28.1451

39. Aoi W, Naito Y, Takagi T, Tanimura Y, Takanami Y, Kawai Y, et al. A novel myokine, secreted protein acidic and rich in cysteine (SPARC), suppresses colon tumorigenesis via regular exercise. Gut. 2013;62:882-9. doi:10.1136/gutjnl-2011-300776

40. Fleming CA, O'Connell EP, Kavanagh RG, O'Leary DP, Twomey M, Corrigan MA, et al. Body Composition, Inflammation, and 5-Year Outcomes in Colon Cancer. JAMA network open. 2021;4:e2115274. doi:10.1001/jamanetworkopen.2021.15274
