# Supplementary material for: DNA methylation of skeletal muscle function‐related secretary factors identifies FGF2 as a potential biomarker for sarcopenia
Source: J Cachexia Sarcopenia Muscle. 2024 Apr 20;15(3):1209–17. doi: 10.1002/jcsm.13472 (PMC11154778; doi:10.1002/jcsm.13472)
Supplement: Supplementary file 7 — Table S3. Primers used for methylation‐specific PCR analysis (MSP) and amplification conditions. [file JCSM-15-1209-s014.docx]

**Supplementary Table 3.** Primers used for methylation-specific PCR analysis (MSP) and amplification conditions.

| **Assay** | **Primer type** | **Primer sequence (5’→3’)** | **Amplicon size, nt** | **Primer annealing temperature, °C** |
| --- | --- | --- | --- | --- |
| ***CTSB_15*** | M-F | GGTTGTAGGTTTTCGGTTGTAGC | 114 | 60 |
|  | M-R | GCAAAAAAACCCGTCTCGAC |  |  |
|  | U-F | GGGTTGTAGGTTTTTGGTTGTAGTG | 123 | 60 |
|  | U-R | CTAAAAACACAAAAAAACCCATCTCA |  |  |
| ***CTSB_17*** | M-F | TTAGTATTTTGGGAGGTCGAGG | 85 | 55 |
|  | M-R | AAAAACGAAATTTCACCGTAT |  |  |
|  | U-F | TAGTATTTTGGGAGGTTGAGG | 89 | 55 |
|  | U-R | TTACTAAAAACAAAATTTCACCATA |  |  |
| ***CXCL12_22*** | M-F | TAGTTTAGTAAAGGTTTGGGGACG | 120 | 60 |
|  | M-R | CTAAACACTAAACTTAAAACCGAAA |  |  |
|  | U-F | TTTAGTAAAGGTTTGGGGATG | 120 | 60 |
|  | U-R | ACCCTAAACACTAAACTTAAAACCAAA |  |  |
| ***FGF19_28*** | M-F | GGTTAGGAGGAATAGTACGGTC | 116 | 60 |
|  | M-R | ACCTAACACCTACCCGAACG |  |  |
|  | U-F | GGGGTTAGGAGGAATAGTATGGTT | 115 | 60 |
|  | U-R | TAACACCTACCCAAACACCA |  |  |
| ***FGF21_59*** | M-F | TAAGTTTTCGTTGTTAGTTCGG | 156 | 55 |
|  | M-R | ACAAATAACAAATCCACAATCCG |  |  |
|  | U-F | TAAGTTTTTGTTGTTAGTTTGG | 157 | 55 |
|  | U-R | TACAAATAACAAATCCACAATCCACT |  |  |
| ***FGF2_30*** | M-F | AGTTTAGGGGATATTCGGGTTTAC | 96 | 60 |
|  | M-R | GTATAATTTCTAACCGCGCGA |  |  |
|  | U-F | AAGTTTAGGGGATATTTGGGTTTAT | 100 | 60 |
|  | U-R | TCCATATAATTTCTAACCACACAAC |  |  |
| ***SESN1_48*** | M-F | GAGGTAAAGTTTTAGGAAAAAGTTTC | 91 | 60 |
|  | M-R | AACCGACAATAACTAACAACCG |  |  |
|  | U-F | GTAAAGTTTTAGGAAAAAGTTTTGT | 90 | 60 |
|  | U-R | ACAACCAACAATAACTAACAACCAC |  |  |
