# Supplementary material for: DNA methylation of skeletal muscle function‐related secretary factors identifies FGF2 as a potential biomarker for sarcopenia
Source: J Cachexia Sarcopenia Muscle. 2024 Apr 20;15(3):1209–17. doi: 10.1002/jcsm.13472 (PMC11154778; doi:10.1002/jcsm.13472)
Supplement: Supplementary file 8 — Table S4. The primers of FGF2_30 for PCR amplification by pyrosequencing. [file JCSM-15-1209-s005.docx]

**Supplementary Table 4.** The primers of FGF2_30 for PCR amplification by pyrosequencing.

FGF2_30

>hg38_dna range=chr4:122827101-122828101 5'pad=500 3'pad=500 strand=- repeatMasking=none

CCTGCTGCAAGGTAGTAATATATTAGCCCCACGTGAGCGATGAGGAAACCGGCTCTGGGGTTAAGCGTCGGCTCAAGGTCTCAAAGCTAGTCACTGCAGGTGGAAGATTAAAGTGTTTGCACTTCTTTTCACTACTGAAACAAAGCTATGGCACTTTATTAACAACATCTGGATTTTTTGCTGGTTGGCTGGTTGGTTTTTGTTTTTGCTCTTTCAGCTTTCGTCAATTCGGAAAAATATAAAACAGAGTGGGTGAGAGTAGTGTGCCATATGCCTCGGGTCGTCCCGAGTCGGAATTTAAGGGCAGGACTCTAGAAAGGTGTTTTTCCAGTAAAGACCGTTTCGTTCTGCTGCGGTCCTTCCTGTCTTTGTCCCCGGGACAGCACGCAGCAGATAGAAGCGGCCAGAGCTGCATGGCGGGGCCGCGGGCATCCCCGACGCGACAGGTTGTCCGCGGGAAGACAGCCAGTCCGCGAGGCCCCGGCGCGCCGCGGCTCCGGGCCGGGGGTACTGGTTTACAGGGCAAATTGGCACACCCCAAAGCCCAGGGGACACCCGGGCCTACGCCGCAGCCCGGAAAGGAGGGGGTGAGTGGTGAACCCGAAACCGCCGAGGGCCGCGCGGCCAGAAACCACACGGAGCGCTAGGGTCGCAGTGAAGATCCGGGAGGAGGGTGCAGGCTGGAGGGGAGAGAGCGGGCGAGAACCCACGAAATGGAAATGAGGCGGAGAGAGCGGGTCGGCACTCACTGTGAGGGTCGCTCTTCTCCCGGACCCCGTCAACTCGGCCGTCGGGGTGGATGCGCAGGAAGAAGCCCCCGTTTTTGCAGTACAGCCGCTTGGGGTCCTTGAAGTGGCCGGGCGGGAAGGCGCCGCTGCCGCCATCCTCGGGCAAGGCGGGCAGCGTGGTGATGCTCCCGGCTGCCATGGTCCCTGCGGGGCCCGGCCGGGATCCCCGAGCCGCTGGAGCCGCGCGGGGAGCCGCCGTCCCCCGGCCCCGGC

Prmier 1

Before conversion：

GGCTCCGGGCCGGGGGTACT

| **Primer Set 1** | | | | **Score: 75 Quality: Medium** | | |
| --- | --- | --- | --- | --- | --- | --- |
| **Primer** | **Id** | | **Sequence** | **Nt** | **Tm, ºC** | **%GC** |
| PCR 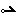 | F1 | | GGAATTTAAGGGTAGGATTTTAGAAAG | 27 | 58.0 | 33.3 |
| PCR 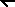 | R1 | | CCCCTAAACTTTAAAATATACCAATTTAC | 29 | 58.4 | 27.6 |
| Sequencing 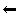 | S1 | | CCAATTTACCCTATAAACC | 19 | 44.0 | 36.8 |
| Target Polymorphisms | Position 34 | | | | | |
| Sequence to Analyze | AATACCCCCR ACCCRAAACC | | | | | |
| Amplicon length | | 259 | | | | |

Prmier 2

Before conversion：

GGACACCCGGGCCTACGCCGCAGCCCGGAAAGGAGGGGGTGAGTGG

| **Primer Set 1** | | | | **Score: 84 Quality: Medium** | | |
| --- | --- | --- | --- | --- | --- | --- |
| **Primer** | **Id** | | **Sequence** | **Nt** | **Tm, ºC** | **%GC** |
| PCR 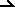 | F1 | | GGGTAGGGGGTATTGGTTTATAGG | 24 | 59.8 | 50.0 |
| PCR 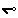 | R1 | | CCACTCACCCCCTCCTTT | 18 | 62.7 | 61.1 |
| Sequencing 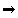 | S1 | | AAATTGGTATATTTTAAAGTTTAGG | 25 | 44.1 | 20.0 |
| Target Polymorphisms | Position35, Position36, Position37, Position38 | | | | | |
| Sequence to Analyze | GGATATTYGG GTTTAYGTYG TAGTTYGGAA AGGAGGGGGT GAGTGGTGAA T | | | | | |
| Amplicon length | | 97 | | | | |

Primer 3

Before conversion：CCGAAACCGCCGAGGGCCGCGCGGCCAGAAACCACACGGAGCGCTAGGGTCGCAGTGAAGATCCGGGAGGAGGGTGCAGGCTGGAGGGG

| **Primer Set 1** | | | | **Score: 85 Quality: Medium** | | |
| --- | --- | --- | --- | --- | --- | --- |
| **Primer** | **Id** | | **Sequence** | **Nt** | **Tm, ºC** | **%GC** |
| PCR 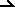 | F1 | | GAGGGGGTGAGTGGTGAA | 18 | 62.4 | 61.1 |
| PCR 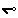 | R1 | | CTCTCTCCCCTCCAACCT | 18 | 60.6 | 61.1 |
| Sequencing 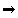 | S1 | | GGGTGAGTGGTGAAT | 15 | 48.4 | 53.3 |
| Target Polymorphisms | Position39, Position40, Position41, Position42, Position43, Position44, Position45, Position46, Position47, Position48 | | | | | |
| Sequence to Analyze | TYGAAATYGT YGAGGGTYGY GYGGTTAGAA ATTATAYGGA GYGTTAGGGT YGTAGTGAAG ATTYGGGAGG AGGGTGTAGG TTGGAGGGG | | | | | |
| Amplicon length | | 114 | | | | |
