# Supplementary material for: DNA methylation of skeletal muscle function‐related secretary factors identifies FGF2 as a potential biomarker for sarcopenia
Source: J Cachexia Sarcopenia Muscle. 2024 Apr 20;15(3):1209–17. doi: 10.1002/jcsm.13472 (PMC11154778; doi:10.1002/jcsm.13472)
Supplement: Supplementary file 9 — Table S5. Characteristics of subjects in the discovery and validation set. [file JCSM-15-1209-s001.docx]

**Supplementary Table 4**. Characteristics of subjects in the discovery and validation set.

| **Variables** | **Discovery set** | | ***P*** | **Validation set 1** | | ***P*** | **Validation set 2** | | ***P*** |
| --- | --- | --- | --- | --- | --- | --- | --- | --- | --- |
|  | **Non-sarcopenia**  **(n=50)** | **Sarcopenia**  **(n=50)** |  | **Non-sarcopenia**  **(n=295)** | **Sarcopenia**  **(n=470)** |  | **Non-sarcopenia**  **(n=141)** | **Sarcopenia**  **(n=123)** |  |
| Age, years | 78.2±4.9 | 78.3±6.9 | 0.908 | 73.0 (72.0, 76.0) | 77.0 (72.0, 81.0) | <0.001 | 76.0 (75.0, 78.0) | 77.0 (70.0, 83.0) | 0.551 |
| Male, *n* (%) | 25 (52.0) | 24 (48.0) | 0.841 | 128 (43.4) | 254 (54.0) | 0.004 | 75 (53.2) | 65 (52.8) | 0.955 |
| BMI, kg/m^2^ | 24.9 (22.9, 26.6) | 19.5 (18.1, 22.1) | <0.001 | 25.1 (22.9, 27.4) | 21.6 (19.7, 23.8) | <0.001 | 24.8 (23.1, 27.4) | 22.6 (20.8, 24.2) | <0.001 |
| Waist hip ratio | 0.91±0.06 | 0.88±0.09 | 0.137 | 0.90 (0.87, 0.95) | 0.89 (0.85, 0.93) | <0.001 | 0.91 (0.87, 0.95) | 0.90 (0.85, 0.94) | 0.061 |
| ASMI, kg/m^2^ | 7.10 (6.50, 7.50) | 4.80 (4.30, 5.50) | <0.001 | 7.00 (6.20, 7.50) | 5.60 (5.20, 6.40) | <0.001 | 7.20 (6.40, 7.90) | 5.60 (5.20, 6.50) | <0.001 |
| Grip strength, kg | 28.2 (24.0, 33.6) | 16.9 (14.5, 21.5) | <0.001 | 25.9 (21.9, 32.0) | 19.9 (15.7 ,24.9) | <0.001 | 30.6 (24.2, 35.9) | 19.8 (15.9, 25.8) | <0.001 |
| Gait speed, m/s | 1.10 (1.05, 1.19) | 0.83 (0.71, 0.90) | <0.001 | 1.10 (1.00, 1.20) | 0.90 (0.80, 0.90) | <0.001 | 1.10 (1.00, 1.20) | 0.90 (0.80, 1.00) | <0.001 |

BMI, body mass index; ASMI, appendicular skeletal muscle mass.
