# Supplementary material for: DNA methylation of skeletal muscle function‐related secretary factors identifies FGF2 as a potential biomarker for sarcopenia
Source: J Cachexia Sarcopenia Muscle. 2024 Apr 20;15(3):1209–17. doi: 10.1002/jcsm.13472 (PMC11154778; doi:10.1002/jcsm.13472)
Supplement: Supplementary file 11 — Table S7. Characteristics of subjects in the pyrosequencing. [file JCSM-15-1209-s013.docx]

**Supplementary Table 7**. Characteristics of subjects in the pyrosequencing.

| Variables | Non-sarcopenia（n=48) | Sarcopenia(n=43) | *P* |
| --- | --- | --- | --- |
| Age, years | 75.6±4.6 | 77.1±6.9 | 0.223 |
| Male, *n* (%) | 24 (50.0) | 21 (48.8) | 0.912 |
| BMI, kg/m^2^ | 25.4 (23.4, 28.2) | 22.8 (20.0, 24.3) | <0.001 |
| Waist hip ratio | 0.93±0.07 | 0.89±0.06 | 0.004 |
| ASMI, kg/m^2^ | 7.16±0.73 | 5.71±0.73 | <0.001 |
| Grip strength, kg | 28.4 (22.8, 35.0) | 18.5 (15.3, 25.3) | <0.001 |
| Gait speed, m/s | 1.10 (1.00, 1.20) | 0.90(0.80, 1.00) | <0.001 |

BMI, body mass index; ASMI, appendicular skeletal muscle mass.
