# Supplementary material for: DNA methylation of skeletal muscle function‐related secretary factors identifies FGF2 as a potential biomarker for sarcopenia
Source: J Cachexia Sarcopenia Muscle. 2024 Apr 20;15(3):1209–17. doi: 10.1002/jcsm.13472 (PMC11154778; doi:10.1002/jcsm.13472)
Supplement: Supplementary file 13 — Table S9. Concordance between the two sarcopenia evaluation methods in the validation population. [file JCSM-15-1209-s008.docx]

**Supplementary Table 9**. Concordance between the two sarcopenia evaluation methods in the validation population.

|  | **Population (n=720)** | **%** |
| --- | --- | --- |
| **Concordant** | 545 | 75.7% |
| Sarcopenic according to both evaluation methods | 336 | 46.7% |
| Non-sarcopenic according to both evaluation methods | 209 | 29.0% |
| **Discordant** | 175 | 24.3% |
| Sarcopenic according to methylation level of FGF2_30 and non-sarcopenic according to AWGS2019 | 100 | 10.4% |
| Non-sarcopenic according to methylation level of FGF2_30 and Sarcopenic according to AWGS2019 | 75 | 13.9% |
