# Supplementary material for: DNA methylation of skeletal muscle function‐related secretary factors identifies FGF2 as a potential biomarker for sarcopenia
Source: J Cachexia Sarcopenia Muscle. 2024 Apr 20;15(3):1209–17. doi: 10.1002/jcsm.13472 (PMC11154778; doi:10.1002/jcsm.13472)
Supplement: Supplementary file 14 — Table S10. Associations of methylation levels of FGF2_30 with the Risk of Sarcopenia. [file JCSM-15-1209-s010.docx]

**Supplementary Table 10.** Associations of methylation levels of FGF2_30 with the Risk of Sarcopenia.

| **Variables** | **Crude OR (95% CI)** | **Adjusted OR (95% CI)** |
| --- | --- | --- |
| Continues | 1.118 (1.100-1.137) ** | 1.106 (1.086-1.126) ** |
| Categorical |  |  |
| ≥0.15 | 1.000 | 1.000 |
| <0.15 | 10.464 (7.776-14.081) ** | 9.223 (6.614-12.861) ** |

The adjusted model included age, sex, and BMI.

OR, odd ratio; CI, confidence intervals; **:*P*<0.001
